# The effect of repeated exposure to ethanol on pre-existing fear memories in rats

**DOI:** 10.1007/s00213-015-4016-9

**Published:** 2015-07-21

**Authors:** Kelvin Quiñones-Laracuente, Marán Y. Hernández-Rodríguez, Christian Bravo-Rivera, Roberto I. Melendez, Gregory J. Quirk

**Affiliations:** Department of Anatomy and Neurobiology, School of Medicine, University of Puerto Rico, PO Box 365067, San Juan, 00936-5067 Puerto Rico; Department of Psychiatry, School of Medicine, University of Puerto Rico, San Juan, Puerto Rico

**Keywords:** Alcohol use disorder, PTSD, Prelimbic, Basolateral amygdala, Central amygdala, c-Fos, Paraventricular thalamus

## Abstract

**Rationale:**

There is a high degree of comorbidity between alcohol use disorder and post-traumatic stress disorder (PTSD), but little is known about the interactions of ethanol with traumatic memories.

**Objectives:**

Using auditory fear conditioning in rats, we asked if repeated exposure to ethanol could modify the retrieval of fear memories acquired prior to ethanol exposure.

**Methods:**

Following auditory fear conditioning, Sprague-Dawley rats were given daily injections of ethanol (1.5 g/kg) or saline over 5 days. Two days later, they were given 20 trials of extinction training and then tested for extinction memory the following day. In a separate experiment, conditioned rats were given repeated ethanol injections and processed for c-Fos immunohistochemistry following a fear retrieval session.

**Results:**

Two days following the cessation of ethanol, the magnitude of conditioned fear responses (freezing and suppression of bar pressing) was significantly increased. This increase persisted the following day. Waiting 10 days following cessation of ethanol eliminated the effect on fear retrieval. In rats conditioned with low shock levels, repeated exposure to ethanol converted a sub-threshold fear memory into a supra-threshold fear memory. It also increased c-Fos expression in the prelimbic prefrontal cortex, paraventricular thalamus, and the central and basolateral nuclei of the amygdala, areas implicated in the retrieval of fear memories.

**Conclusions:**

These results suggest that repeated exposure to ethanol may exacerbate pre-existing traumatic memories.

## Introduction

People with post-traumatic stress disorder (PTSD) are approximately three times more likely to develop alcohol use disorder than people without PTSD (Kessler et al. 1997; McCarthy and Petrakis 2010). Comorbidity of PTSD and alcohol use disorder could be due to the anxiolytic properties of ethanol, which is often used to “self-medicate” (Adams 1886; Fletcher et al. 2010). In support of this idea, symptoms of PTSD and alcohol use disorder are positively correlated (Ouimette et al. 2010), and PTSD has been shown to predict the emergence of alcohol use disorder following a traumatic experience (McFarlane et al. 2009). Another possibility, however, is that alcohol use contributes to PTSD symptoms by exacerbating anxiety.

Rodent studies of fear conditioning have been useful for assessing the effects of ethanol on fear and anxiety. In support of an anxiolytic role, acute injections of ethanol impaired conditioned fear retrieval (Baum 1969; Lattal 2007), as well as its acquisition (Broadwater and Spear 2013a). However, studies examining the effects of repeated ethanol injections over several days are more relevant for alcohol use disorders. In support of an anxiogenic effect, multiple days of ethanol exposure followed by a withdrawal period increased the acquisition of conditioned fear (Bertotto et al. 2006) or impaired extinction (Holmes et al. 2012). This suggests that repeated ethanol could compromise extinction-based therapies for anxiety disorders. Few studies, however, have assessed the effects of repeated ethanol on fear memories acquired *prior to* ethanol exposure, resembling the use of ethanol as self-medication.

We therefore examined the effects of repeated ethanol injections in rats on the retrieval of conditioned fear memories acquired prior to ethanol exposure. Fear memory was tested 3 days after the last injection, to avoid intoxication and the majority of withdrawal effects (Livy et al. 2003). c-Fos immunohistochemistry was used to assess the effects of repeated ethanol on the conditioned fear circuit. We found that repeated ethanol administration augmented fear memories and increased activity in prefrontal-thalamic-amygdala areas implicated in fear retrieval.

## Materials and methods

### Subjects

A total of 97 male Sprague-Dawley rats (Harlan Laboratories, Indianapolis, IN) weighing 270–320 g were housed, one per cage, and handled as previously described (Quirk et al. 2000). Rats were restricted to 18 g/day of standard laboratory rat chow and trained to press a bar for food on a variable interval schedule of reinforcement (VI-60). Pressing a bar for food ensured a constant level of movement against which freezing can be reliably measured during long training sessions. All procedures were approved by the Institutional Animal Care and Use Committee of the University of Puerto Rico, School of Medicine, in compliance with National Institutes of Health guidelines for the care and use of laboratory animals.

### Fear conditioning

Rats were fear conditioned in standard experimental chambers (27 cm long, 28 cm wide, 28 cm tall; Coulbourn Instruments, Allentown, PA) located inside sound-attenuating cubicles (Med Associates, Burlington, VT), similar to our previous studies (Sierra-Mercado et al. 2011). The floor of the chambers consisted of stainless steel bars that delivered a scrambled electric footshock. The same chamber was used for conditioning, extinction training, and retrieval tests. Rats were conditioned with a pure tone (30 s, 4 kHz, 75 dB) that co-terminated with shock delivery to the floor grids (0.5 s, 0.20 or 0.50 mA). All trials were separated by a variable interval averaging 3 min. Throughout all phases of training, rats pressed a lever for a sucrose pellet on a VI-60 schedule.

Fear conditioning consisted of one habituation tone (no shock), followed by six tone-shock pairings. One day later, 20 tones (3 min ITI) without shock were presented to extinguish the conditioning association. Twenty-four hours after extinction training, we tested the extinction memory with an eight-tone test. A separate group of rats was conditioned using a sub-threshold conditioning shock (0.2 mA) and was tested with a two-tone test, instead of an eight-tone test, to avoid extinguishing freezing.

### Ethanol administration

A 30 % (vol/vol) ethanol solution was prepared by diluting a 95 % ethanol stock solution with 0.9 % saline. Rats received injections of saline or 1.5 g/kg ethanol i.p. once daily for 1 day or 5 consecutive days in their home cage, as previous studies have done (Lattal 2007). Blood-ethanol concentrations have been shown to peak at 200 mg/dl 20 min after i.p. injection (Spirduso et al. 1989). Repeated ethanol was administered 1 day after conditioning. Five days of ethanol treatment is sufficient to induce significant alterations in processes underlying synaptic plasticity, including glutamate transporter function and glutamate release (Kapasova and Szumlinski 2008; Melendez et al. 2006). Following the last injection, rats remained drug-free for 2 days and then were tested on the third day in the absence of drug.

### Immunohistochemistry

Immunohistochemistry was performed as described previously (Bravo-Rivera et al. 2015). At the end of a two-tone retrieval test, a subset of four saline-treated and four repeated ethanol-treated rats was perfused transcardially with 200 ml of 0.9 % saline followed by 500 ml of 4 % paraformaldehyde (PFA) in 0.1 M phosphate buffer (PBS) at pH 7.4. The brains were removed and fixed overnight in 4 % PFA and transferred to 30 % sucrose in 0.1 M PBS for 48 h, for cryoprotection. Frozen sections were cut coronally (40 μm) with a cryostat (CM 1850; Leica) at different levels of the medial prefrontal cortex (mPFC), amygdala, and thalamus.

Sections were initially blocked in a solution of 2 % normal goat serum (NGS, Vector Laboratories, USA) plus 0.1 % tween (Tween-20, Sigma-Aldrich, USA) in 0.1 M PBS for 1 h. Afterwards, sections were incubated overnight at room temperature with anti-c-Fos serum raised in rabbit (Ab-5, Oncogene Science, USA) at a concentration of 1:20,000. The primary antiserum was localized using a variation of the avidin-biotin complex system. Sections were then incubated for 2 h at room temperature in a solution of biotinylated goat anti-rabbit IgG (Vector Laboratories) and placed in a mixed avidin-biotin horseradish peroxidase complex solution (ABC Elite Kit, Vector Laboratories) for 90 min. Black/brown immunoreactive nuclei labeled for c-Fos were visualized after 15 min of exposure to a solution containing 0.02 % diaminobenzidine tetrahydrochloride with 0.3 % nickel ammonium sulphate in 0.05 M Tris buffer, pH 7.6, followed by a 10-min incubation period in a chromogen solution with glucose oxidase (10 %) and d-glucose (10 %). The reaction was stopped using 0.1 M PBS (pH 7.4). Sections were mounted on gelatin-coated slides, dehydrated, and cover slipped. Counter sections were collected, stained for Nissl bodies, cover slipped, and used to determine the anatomical boundaries of each structure analyzed.

c-Fos-immunoreactive neurons were counted at ×20 magnification with an Olympus microscope (Model BX51) equipped with a digital camera. Micrographs were generated for prelimbic cortex (PL, +3.00 to 3.70 AP), infralimbic cortex (IL, +3.00 to 3.70 AP), basolateral nucleus of the amygdala (BLA, −3.00 to −2.00 AP), central nucleus of the amygdala, divided into lateral (CeL, –3.00 to −2.00 AP) and medial (CeM, −3.00 to −2.00 AP) portions, and the paraventricular nucleus of the thalamus (PVT, −3.00 to −2.00 AP). c-Fos-positive cells were automatically counted and averaged for each hemisphere in 2–3 different sections for each structure (Metamorph software version 6.1). Density was calculated by dividing the number of c-Fos-positive neurons by the total area of each region.

### Data collection and analysis

Behavior was recorded with digital video cameras (Micro Video Products, Bobcaygeon, Ontario, Canada), and freezing was measured using commercially available software (FreezeScan, Clever Systems, Reston, VA, USA). Tone-induced suppression of bar pressing was calculated as a suppression ratio as follows: (pretone − tone)/(pretone + tone). A value of 0 indicates no suppression, while a value of 1 indicates complete suppression. Trials were averaged in blocks of two and compared with repeated-measures two-way ANOVAs. c-Fos counts per brain structure were compared with Student’s *t* tests (two-tailed). Data were analyzed with STATISTICA 6 (StatSoft, Inc).

## Results

### Repeated ethanol exposure strengthens previously acquired fear memories

**Fig. 1:**
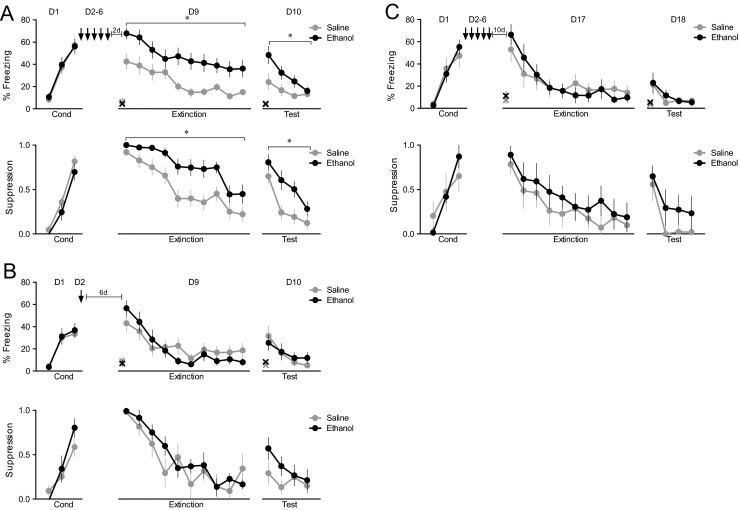
Repeated, but not acute, injection of ethanol increases retrieval of previously acquired fear memory. a (*top*) Rats were conditioned on day 1 (*D1*) and given 5 days of repeated ethanol injections (*D2-6*). After 2 days drug-free, extinction training and extinction testing were given on days 9 and 10 (*D9-10*). **a** (*bottom*) Same animals as in (**a**), *top*, showing suppression of bar pressing to the tone. *n* = 14 per group. **b** (*top*) Rats were conditioned on day 1 (*D1*), and the next day, received only one injection of saline or ethanol (*D2*). After 6 days drug-free, extinction training was given on day 9 (*D9*) and extinction test on day 10 (*D10*). **b** (bottom) Same animals as in (**b**), *top*, showing suppression of bar pressing to the tone. *n* = 18 per group. **c** (*top*) Rats were conditioned on day 1 (*D1*) and then were injected with saline or ethanol over the next 5 days (*D2-6*). After 10 days drug-free, rats were given extinction training on day 17 (*D17*) and extinction test on day 18 (*D18*). **c** (bottom) Same animals as in (**c**), top, showing suppression of bar pressing to the tone. *p < 0.05, *n* = 10 per group. Bars indicate SEM. *X* indicates freezing during the 30 s prior to the first tone, ↓: daily injection of saline or ethanol

Rats were fear conditioned to a tone paired with a footshock on day 1 and, for the next 5 days, were given an injection (one per day) of saline or ethanol (days 2–6), followed by 2 drug-free days (days 7–8) (Fig. 1a, top). On the following day (day 9), rats were given 20 extinction trials (tones with no shock). Repeated exposure to ethanol increased freezing to the previously conditioned tone throughout the session, starting in the first trial block. Repeated-measures ANOVA revealed a main effect of group (*F*_(1,34)_ = 11.08, *p* = 0.002) and trial block (*F*_(9,306)_ = 13.62, *p* < 0.001), but no group × trial block interaction (*p* = 0.76). Tone-induced suppression of bar pressing (Fig. 1a, bottom) was similarly elevated, showing a main effect of group (*F*_(1,34)_ = 19.45, *p* < 0.001), trial block (*F*_(9,306)_ = 21.86, *p* < 0.001), and a group × trial block interaction (*F*_(9,306)_ = 8.58, *p* < 0.001). Prior to the first tone, there was no difference in presses per minute (saline 9.5 ± 2.4, ethanol 11.1 ± 2.9, *p* = 0.89), or freezing (saline 6.7 ± 3.3 %, ethanol 4.6 ± 2.2 %, *p* = 0.48). Thus, repeated exposure to ethanol increased the retrieval of conditioned fear throughout the extinction session.

In an extinction test session given the following day (day 10), ethanol rats again exhibited increased freezing in the first trial block (Fig. 1a, top). Repeated-measures ANOVA revealed a main effect of group (*F*_(1,34)_ = 7.49, *p* = 0.01), trial block (*F*_(3,102)_ = 8.71, *p* < 0.001), but not a group × trial block interaction (*p* = 0.14). For suppression, repeated-measures ANOVA revealed a main effect of group (*F*_(1,34)_ = 5.95, *p* = 0.02) and trial block (*F*_(3,102)_ = 31.75, *p* < 0.001), but not a group × trial block interaction (*p* = 0.15, Fig. 1a). Prior to the first tone, there was no difference in presses per minute (saline 11.4 ± 1.9, ethanol: 10.2 ± 2.1, *p* = 0.78), or freezing (saline 5.3 ± 1.7 %, ethanol 4.2 ± 2.0, p = 0.76).

To determine if ethanol’s effect on fear memory requires repeated injections, we conditioned a separate group of rats on day 1 and administered a single injection of saline or ethanol on day 2 (Fig. 1b). We then waited 6 days prior to testing (day 9), to match the delay in our first experiment. In contrast to repeated injections, a single injection of ethanol induced no group differences during extinction training (*F*_(1,26)_ = 0.10, *p* = 0.75) or extinction recall test (*F*_(1,26)_ = 0.07, *p* = 0.80). There were also no significant group differences in suppression (extinction training: *p* = 0.51; extinction recall: *p* = 0.08). Prior to the first tone, there was no difference in the presses per minute (saline 13.4 ± 2.8, ethanol 14.2 ± 3.7, *p* = 0.88), or freezing (saline 9.2 ± 3.6 %, ethanol 7.0 ± 3.0 %, p = 0.75). Prior to the first tone in extinction recall, there was no difference in presses per minute (saline 14.7 ± 3.1, ethanol 14.3 ± 3.9, *p* = 0.92), or freezing (saline 8.3 ± 2.3 %, ethanol 5.4 ± 1.6 %, p = 0.32). Thus, repeated exposure to ethanol is necessary for the increased fear retrieval observed in the first experiment.

### The effect of repeated ethanol on fear is time limited

We next determined if the exacerbating effects of ethanol on fear memories would persist past 3 days following cessation of ethanol, in light of reports that withdrawal effects can exceed 3 days (Veatch 2006). We therefore repeated the experiment, but tested rats 10 days following cessation of ethanol treatment (Fig. 1c). In contrast to our previous results, repeated ethanol injections followed by 10 drug-free days had no effect on fear retrieval. Repeated-measures ANOVA of freezing yielded no effect of group (*p* = 0.98) nor group × trial block interaction (*p* = 0.46). Similarly, bar-press suppression showed no effect of group (*p* = 0.46) nor group × trial block interaction (*p* = 0.94). Prior to the first tone, there was no difference in presses per minute (saline 12.4 ± 3.5, ethanol 10.7 ± 3.1, *p* = 0.69), or freezing (saline 7.2 ± 3.1 %, ethanol 11.4 ± 3.7 %, p = 0.74). On the following day (day 18), the two groups continued to show equivalent levels of freezing (*p* = 0.77) and suppression (*p* = 0.20). Prior to the first extinction recall tone, there was also no difference in presses per minute (saline 13.0 ± 3.2, ethanol 11.6 ± 3.3, *p* = 0.65), or freezing (saline 3.4 ± 0.5 %, ethanol 5.6 ± 1.1 %, p = 0.21). Thus, the memory effects of repeated ethanol injections last 3 days, but not 10 days.

### Repeated ethanol exposure enhances the retrieval of new fear memories

**Fig. 2:**
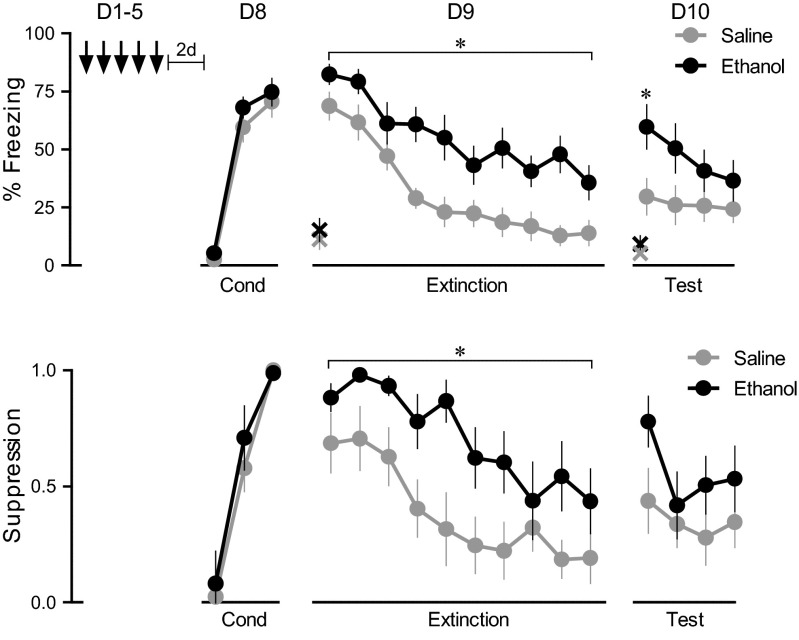
Repeated ethanol given prior to conditioning increases retrieval of well-consolidated fear memory. Rats received 5 days of saline or ethanol injections followed by conditioning (*D8*) (*top*). Extinction training was given on day 9 (*D9*), and an extinction test was given on day 10 (*D10*). Same animals as in (**a**), showing suppression of bar pressing to the tone. *p < 0.05, *n* = 13 per group. *Bars* indicate SEM. *X* indicates the freezing 30 s before the first tone, ↓: daily injection of saline or ethanol

Repeated exposure to ethanol augmented the retrieval of a previously acquired fear memory. In the next experiment, we asked whether administering repeated ethanol prior to conditioning would affect the acquisition of fear memory. Naïve rats received 5 days of ethanol or saline injections, followed by 3 days drug-free. On the conditioning day (day 8), saline and ethanol groups showed similar levels of conditioning (Fig. 2). The following day (day 9), however, rats treated with ethanol exhibited increased freezing throughout the 20 trial extinction session (day 9). Repeated-measures ANOVA revealed a main effect of group (*F*_(1,24)_ = 11.40, *p* < 0.001) and trial block (*F*_(9,216)_ = 29.15, *p* < 0.001), but no group × trial block interaction (*p* = 0.15). For suppression, there was a main effect of group (*F*_(1,24)_ = 7.31, *p* < 0.001) and trial block (*F*_(9,216)_ = 9.80, *p* < 0.001), but no group × trial block interaction (*p* = 0.51). Prior to the first tone, there was no difference in presses per minute (saline 8.9 ± 1.4, ethanol 7.8 ± 2.2, *p* = 0.45). Both groups significantly extinguished their levels of freezing (*p* < 0.001) and suppression (*p* < 0.001), or freezing (saline 11.3 ± 4.2 %, ethanol 15.3 ± 5.0 %, p = 0.97). The following day (day 10), ethanol rats continued to express increased freezing in block 1 (saline 30 %, ethanol 60 %; *t*_(24)_ = −2.41, *p* = 0.023). Prior to the first extinction recall tone, there was no difference in presses per minute (saline 10.4 ± 3.3, ethanol 10.1 ± 2.7, *p* = 0.75), or freezing (saline 9.3 ± 3.5 %, ethanol 5.3 ± 1.4 %, p = 0.44). Thus, repeated ethanol enhances retrieval of consolidated fear memories acquired either before or after ethanol exposure.

### Repeated ethanol increases c-Fos expression in the prefrontal cortex, thalamus, and amygdala

**Fig. 3:**
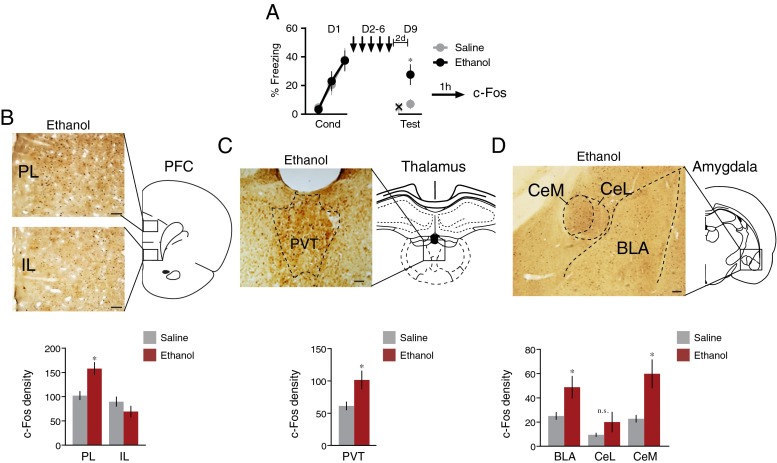
Repeated ethanol maintained the retrieval of a sub-threshold fear memory and increased c-Fos expression. **a** Rats were conditioned with a weak shock (0.2 mA) on day 1 (*D1*), followed by 5 days of saline or ethanol injections (*D2-6*). On day 9 (*D9*), there was a fear retrieval test (*n* = 13 for saline rats and *n* = 14 for ethanol rats). A subset of rats (*n* = 4 per group) were sacrificed for c-Fos immunohistochemistry 1 h after test. **b** An example of the c-Fos expression in the prefrontal cortex, with group data below. *Scale bar*, 0.1 mm. **c** An example of the c-Fos expression in the paraventricular thalamus, with group data below. *Scale bar*, 0.1 mm. **d** An example of the c-Fos expression in the amygdala, with group data shown below. *Scale bar*, 0.2 mm. *p < 0.05, *t* test. *Bars* indicate SEM. *X* indicates the freezing 30 s before the first tone, ↓: daily injection of saline or ethanol. *PL* prelimbic cortex, *IL* infralimbic cortex, *PVT* paraventricular thalamus, *CeM* central amygdala (*medial part*), *CeL* central amygdala (*lateral part*), *BLA* basolateral amygdala

We next determined if the retrieval-enhancing effect of repeated ethanol could convert sub-threshold fear memories into supra-threshold ones. Rats were conditioned with a weak shock (0.2 mA), injected for 5 days with saline or ethanol, and then tested 3 days later (Fig. 3a). Saline-injected rats showed virtually no freezing at test, whereas ethanol-injected rats showed moderate freezing at test (saline 12 %, ethanol 35 %; *t*_(26)_ = −2.61, *p* = 0.015). Prior to the first retrieval tone, there was no difference in freezing (saline 4.3 ± 2.5 %, ethanol 5.2 ± 1.7 %, p = 0.95).

One hour following the test tones, a subgroup of rats from each group was sacrificed and their brains harvested for c-Fos labeling. Examples of c-Fos labeling in the prefrontal cortex, paraventricular thalamus, and amygdala are shown in Fig. 3b–d. In the prefrontal cortex (Fig. 3b), ethanol-treated rats showed significantly more c-Fos labeling than saline-treated rats in the prelimbic cortex (saline 102 counts/0.1 mm^2^, ethanol 158 counts/0.1 mm^2^; *t*_(7)_ = −4.25, *p* = 0.004), but not the infralimbic cortex (saline 90 counts/0.1 mm^2^, ethanol 69 counts/0.1 mm^2^; *t*_(7)_ = 1.59, *p* = 0.16). The paraventricular nucleus of the thalamus (PVT), a region necessary for retrieval of fear memories 1–7 days after fear conditioning (Do-Monte et al. 2015; Padilla-Coreano et al. 2012) also showed higher levels of c-Fos in ethanol-treated rats (Fig. 3c) (saline 61 counts/0.1 mm^2^, ethanol 102 counts/0.1 mm^2^; *t*_(7)_ = −2.68, *p* = 0.03). In the amygdala (Fig. 3d), ethanol-treated showed had significantly more c-Fos labeling in the basolateral amygdala (BLA) (saline 25 counts/0.1 mm^2^, ethanol 49 counts/0.1 mm^2^; *t*_(7)_ = −2.42, *p* = 0.045), the medial subdivision of the central nucleus (CeM) (saline 23 counts/0.1 mm^2^, ethanol 60 counts/0.1 mm^2^; *t*_(7)_ = −3.54, *p* = 0.0094), but not in the lateral subdivision (CeL) (saline 10 counts/0.1 mm^2^, ethanol 20 counts/0.1 mm^2^; *t*_(7)_ = −1.46, *p* = 0.19). Thus, repeated exposure to ethanol increased activity in prefrontal-thalamic-amygdalar nuclei implicated in retrieval of consolidated fear memories (Do-Monte et al. 2015).

## Discussion

We observed that repeated exposure to ethanol augmented the retrieval of previously acquired fear memories. When given prior to conditioning, repeated ethanol increased fear retrieval 24 h after conditioning, but not on the day of conditioning, suggesting that ethanol’s effects were specific for retrieval of consolidated fear memories. Moreover, repeated ethanol converted sub-threshold fear memories to supra-threshold memories and increased activity in PL, PVT, BLA, and CeM, areas implicated in retrieval of conditioned fear.

It is possible that the increased fear responses following repeated ethanol may be due to the stress associated with repeated exposure to ethanol (Przybycien-Szymanska et al. 2011; Varlinskaya and Spear 2014; Willey et al. 2012). This is unlikely, however, because repeated exposure to a stressor has been shown to increase freezing during the acquisition phase of fear conditioning (Farrell et al. 2010), whereas our pre-training ethanol injections did not. It is also unlikely that the effects are due to acute withdrawal effects, as rats were tested 3 days after the final injection, when acute withdrawal effects are no longer present (Bertotto et al. 2006; Koob 2004).

In most prior studies assessing the effects of repeated ethanol, rats were exposed to ethanol prior to conditioning. In agreement with our pre-training ethanol findings, repeated, but not acute, exposure to ethanol has been shown to increase subsequent contextual fear conditioning (Bertotto et al. 2006; Broadwater and Spear 2013b). Using auditory fear conditioning, Holmes and colleagues observed that 20 days of repeated ethanol exposure had no effect on acquisition or extinction, but impaired recall of extinction memory (Holmes et al. 2012). Despite some methodological differences, there appears to be a growing consensus that pre-training ethanol exposure increases subsequent fear learning. The main question of our study, however, was whether repeated ethanol modifies retrieval of previously acquired fear memories. Prior studies have not examined the effects of post-training ethanol on auditory fear conditioning, but post-training ethanol was shown to increase retrieval of contextual fear conditioning in mice (Quadros et al. 2003) and impair retrieval of trace fear conditioning (Hunt et al. 2009).

The ethanol-induced increase in fear retrieval was present when rats were tested 3 days, but not 10 days, after cessation of repeated ethanol. This timing of effects suggests the possibility of an extended withdrawal phase that is present 3 days after cessation of ethanol. Bertotto et al. (2006) observed normal locomotion and anxiety levels 3 days after chronic ethanol exposure; however, rapid eye movement sleep at this timepoint is still elevated (Veatch 2006) which could enhance memory consolidation (Datta and O'Malley 2013).

Consistent with the increased retrieval of previously learned fear, we observed increased c-Fos activity in PL, PVT, and CeM, areas implicated in retrieval of conditioned fear (Do-Monte et al. 2015; LeDoux et al. 1988; Pape and Pare 2010; Penzo et al. 2015; Sierra-Mercado et al. 2011; Sotres-Bayon and Quirk 2010). We recently reported that the PL-PVT-CeA circuit is recruited into the fear retrieval circuit 1–7 days after fear conditioning (Do-Monte et al. 2015). This is consistent with the findings of the present study, as repeated ethanol increased retrieval of fear either 1 or 7 days after fear conditioning, but not during conditioning itself (Fig. 3). Our present findings are also consistent with our previous observation that the PL-PVT-CeA circuit is necessary for long-term maintenance of the fear memory (Do-Monte et al. 2015), as rats receiving ethanol could retain a sub-threshold memory for 7 days. An important caveat is that these c-Fos findings do not distinguish a direct effect of ethanol on these circuits from an indirect effect via increased expression of freezing to the tone.

The increase in c-Fos in amygdala central nucleus (CeA) is interesting in light of the well-documented role of the CeA in ethanol addiction (Koob 2004; Nie et al. 2004; Roberto et al. 2004). Repeated (but not acute) ethanol increases glutamate release in CeA and sensitizes the response of NMDA receptors in CeA to subsequent ethanol (Roberto et al. 2004). CeM projects to hypothalamic and midbrain areas controlling fear responses (Hitchcock and Davis 1987; LeDoux et al. 1988; McDannald 2010). Thus, increases in fear responses may develop alongside alcohol use disorder, thereby contributing to comorbidity with PTSD.

Further studies using ethanol self-administration models are needed to fully understand the effects of ethanol on pre-existing fear memories. Indeed, voluntary ethanol consumption more closely resembles the human condition and can differ from systemic administration in terms of behavioral effects (Freund 1975; Goldstein 1975). The amount of ethanol voluntarily consumed by rats increases following contextual fear conditioning (Meyer et al. 2013), which according to our findings would further augment fear retrieval. It would also be interesting to vary the number of days between fear conditioning and ethanol administration (1 day in our study), to model remote fear memories that are often associated with PTSD.

An increasing number of studies have reported high rates of comorbidity between PTSD and alcohol use disorder (Back et al. 2006; Dedert et al. 2009; Freeman and Kimbrell 2004; McCarthy and Petrakis 2010; McDevitt-Murphy et al. 2010; Ray et al. 2009). Our results suggest that long-term alcohol use could exacerbate PTSD by increasing fear retrieval and converting non-traumatic memories into traumatic memories. Increased fear could compromise extinction-based exposure therapy by making it more stressful, thereby increasing the rate of dropout (Hembree et al. 2003), or by impairing the extinction process (Holmes et al. 2012). For PTSD patients who suffer from alcohol use disorder, our findings support previous recommendations that alcohol use be terminated prior to initiating exposure therapy (Becker et al. 2004; Pitman et al. 1991). In fact, a recent study found that combining exposure therapy with treatment for alcohol use disorder improved prognosis (Mills et al. 2012).
